# Triage of Amyotrophic Lateral Sclerosis Patients during the COVID-19 Pandemic: An Application of the D50 Model

**DOI:** 10.3390/jcm9092873

**Published:** 2020-09-05

**Authors:** Robert Steinbach, Tino Prell, Nayana Gaur, Beatrice Stubendorff, Annekathrin Roediger, Benjamin Ilse, Otto W. Witte, Julian Grosskreutz

**Affiliations:** 1 Hans Berger Department of Neurology, Jena University Hospital, 07747 Jena, Germany; Tino.Prell@med.uni-jena.de (T.P.); Nayana.Gaur@med.uni-jena.de (N.G.); Beatrice.Stubendorff@med.uni-jena.de (B.S.); Annekathrin.Roediger@med.uni-jena.de (A.R.); Benjamin.Ilse@med.uni-jena.de (B.I.); Otto.Witte@med.uni-jena.de (O.W.W.); Julian.Grosskreutz@med.uni-jena.de (J.G.); 2 Center for Healthy Ageing, Jena University Hospital, 07747 Jena, Germany

**Keywords:** amyotrophic lateral sclerosis, disease progression, D50, COVID-19, SARS-CoV-2, triage, gastrostomy, assisted ventilation

## Abstract

Amyotrophic lateral sclerosis (ALS) is a progressive neuromuscular disease, the management of which requires the continuous provision of multidisciplinary therapies. Owing to the novel coronavirus disease (COVID-19) pandemic, regular contact with ALS patients at our center was severely restricted and patient care was at risk by delay of supportive therapies. We established a triage system based on the D50 disease progression model and were thus able to identify a prospective cohort with high disease aggressiveness (D50 < 30). Thirty-seven patients with highly aggressive disease were actively offered follow-up, either via telephone or on-site, depending on their disease-specific needs and abilities. We describe here the procedures, obstacles, and results of these prescient efforts during the restrictions caused by COVID-19 in the period between March and June 2020. In conclusion, four patients with highly aggressive disease were initiated with non-invasive ventilation and two received a gastrostomy. We could show that a comparable amount of advanced care was induced in a retrospective cohort within a similar time period one year prior to the COVID-19 outbreak. Our workflow to identify high-risk patients via D50 model metrics can be easily implemented and integrated within existing centers. It helped to maintain a high quality of advanced care planning for our ALS patients.

## 1. Introduction

Amyotrophic lateral sclerosis (ALS) is a fatal neurodegenerative disease characterized by the predominant loss of motor neuron function. The mean survival time is three years after symptom onset, with most patients succumbing to respiratory insufficiency [1,2]. However, high inter-individual heterogeneity has been reported for ALS progression and survival rates [3,4]. Despite continuous efforts to develop curative therapies, the only globally approved therapy, Riluzole, was discovered over two decades ago [5,6]. Patient care entails extensive symptomatic and supportive therapies throughout the disease course and requires constant patient counselling and allied health care [7,8,9]. In keeping with this, an increased number of tertiary centers provide individualized multidisciplinary care for patients with ALS [7,10].

The rapid outbreak of the novel coronavirus disease (COVID-19) pandemic in early 2020 brought restrictions and disruptions to public life and substantially impacted the healthcare system. National and local governments undertook unprecedented measures, including banning elective hospitalization, reallocating resources and staff to intensive care units and limiting public transportation. This caused a severe breakdown of the established care workflow for patients in our ALS center. In particular, planned hospitalizations for the evaluation of the necessity and/or initiation of assisted ventilation and/or gastrostomies were indefinitely postponed. Advanced care planning managed via three monthly appointments was affected owing to social distancing and quarantine requirements, travel restrictions, and patients’ own apprehensions and cancellations. We had to greatly reduce in- and out-patient consultations whilst ensuring timely critical ALS-specific therapies.

Thus, a triage system needed to be promptly established. Broadly speaking, there are two extremes of the ALS disease spectrum that need to be differentially catered to. The first encompasses patients with highly aggressive disease whose deterioration occurs within weeks or even days, e.g., the onset of orthopnea or dyspnea. Herein, the disruption of symptomatic treatment could directly lead to the need for emergency hospitalization. In the second scenario, patients suffer from a slowly progressive disease with muscular dysfunction occurring over weeks and months rather than days. Herein, postponing specialized follow-ups is unlikely to lead to immediate dire consequences assuming that the guiding principles of preventative counselling and care planning have been adhered to in prior appointments.

To provide a risk-adapted triage for the highly heterogenous cohort of our ALS patients, we used the recently developed D50 disease progression model [11,12,13]. Doing so enabled us to identify patients with higher disease aggressiveness who required special attention and more frequent follow-ups at the peak of the COVID-19 outbreak in Germany and the ensuing period of severe restrictions of public life and elective health care provisions. We defined two primary outcome goals for the patients: 1) the appropriate initiation of assisted ventilation that is known to increase survival and improve quality of life [14,15], and 2) the implantation of a feeding tube (gastrostomy), early enough in the disease course which improves outcome [16,17,18,19].

## 2. Methods

### 2.1. Design and Study Cohorts

**Figure 1:**
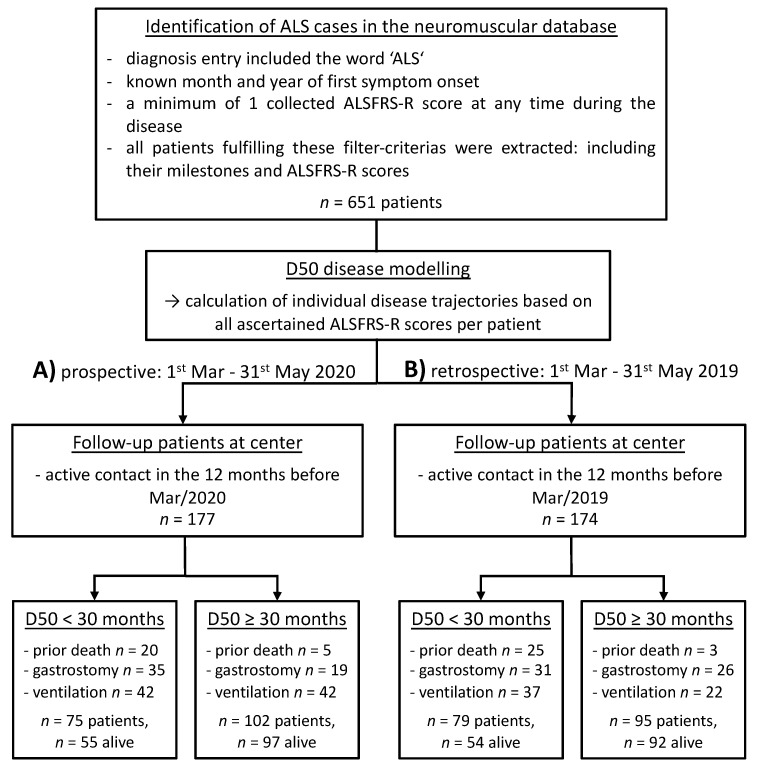
Consort diagrams for A) the prospective and B) the retrospective cohort under active follow-up. Patients were identified based on milestone-related entries in the neuromuscular database. Patients were then stratified based on their individual D50 values as either having high disease aggressiveness (D50 < 30 moths) or low aggressiveness (D50 ≥ 30 months). In the bottom-last row the numbers of events that occurred before the cut-off day (1^st^ March) are given for prior death, gastrostomy or initiation of assisted ventilation. *Abbreviations:* ALS = Amyotrophic Lateral Sclerosis; ALSFRS-R = ALS Functional Rating Scale (Revised); D50 = estimated time in months for an individual to lose 50% of functionality.

We performed analyses in two independent cohorts of patients at the neuromuscular center of the Jena University hospital (Figure 1 displays a CONSORT diagram). For the prospective cohort we selected patients who had at least one face-to-face appointment within the 12 months prior to 1^st^ March 2020 (*n* = 177; Figure 1A). For these patients we applied the proposed triage system based on the D50 model. Events that occurred between 1^st^ March and 31^st^ May 2020 in this cohort were subsequently analyzed. For comparison purposes, we identified a second cohort of patients who were treated in a similar time frame one year before the COVID-19 pandemic (1^st^ March until 31^st^ May 2019; Figure 1B).

Patient-specific data were extracted from a center specific neuromuscular database developed by J.G. (currently Microsoft^®^ Access^®^ 2010 v14.0, Seattle, WA, USA) for both cohorts including the following: age, gender, revised ALS Functional Rating Scales (ALSFRS-R), time-point and region of first symptom onset (bulbar, limb, generalized or respiratory), time of gastrostomy, time of start of ventilatory support (invasive or non-invasive), and time of death.

The procedures conducted for the purpose of this study have been previously approved by the local Ethics committee (Nr 3633-11/12).

### 2.2. The D50 Disease Progression Model

The D50 model provides unified parameters of patients’ individual disease course, e.g., of overall disease aggressiveness (D50) or individual disease covered (rD50) [11,12,13]. Calculation of the model is based on the revised ALS functional rating scale (ALSFRS-R), the internationally used scale to quantify the remaining muscle functions of patients affecting their daily life [20].

**Figure 2:**
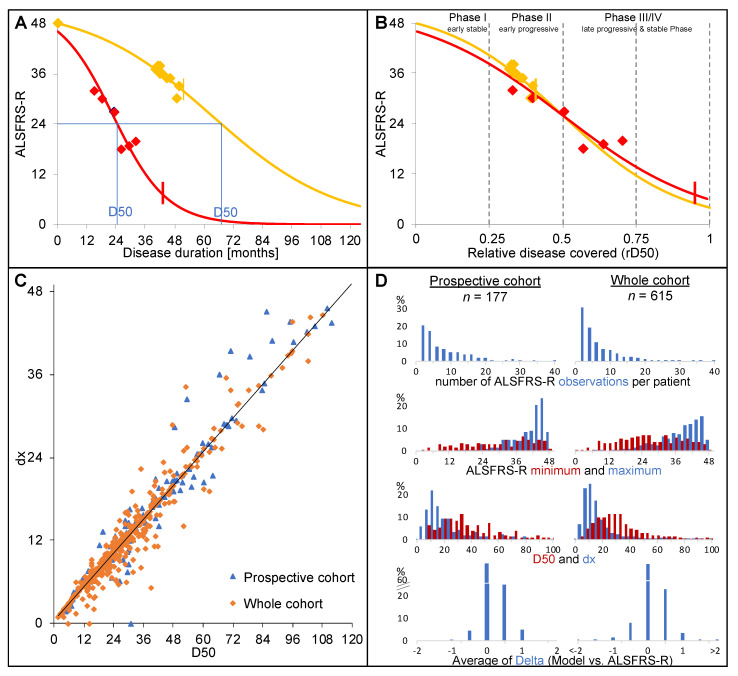
D50 disease progression model. (**A**) Patient with high (depicted in red, D50 = 22.8 months) and another with lower disease aggressiveness (depicted in yellow, D50 = 63.3 months). The individual disease curve is modelled based on all available ALSFRS-R scores (squares). The vertical lines mark the acute position in the disease course at March 2020. (**B)** Normalization with rD50, which describes individual disease course covered in reference to D50, allows for comparability between patients who all proceed through similar Phases (I-IV) of functional decline, despite vastly differing disease aggressiveness. (**C**) D50 and dx (time constant of ALSFRS-R total score decline) correlate linearly in all 651 patients who were available in the database and the prospective cohort respectively. (**D**) Modelling data for the prospective cohort (left column) and for all ALS patient data available at our neuromuscular center. From top to bottom, the number of obtained ALSFRS-R scores per patient, the minimum ALSFRS-R score (red), the maximum ALSFRS-R score (blue), and the calculated D50 (red) and dx (blue) values, and the offset between model data and scores do not differ between the cohorts.

Briefly, the D50 model describes the disease course of individual ALS patients as a sigmoidal state transition from full health to functional loss (Figure 2A). The value dx describes the time constant of ALSFRS-R total score decline and the value D50 is defined as the estimated time taken in months for a patient to lose 50% of his/her functionality (equivalent to an ALSFRS-R total score of 24). dx and D50 correlated linearly in this (Figure 2B) and other ALS cohorts [11,13]. Thus, the D50 value alone provides a unified descriptive measure of individual patients’ overall disease aggressiveness. This allowed us to classify patients as having either low (D50 ≥ 30 months) or high (D50 < 30 months) disease aggressiveness. A normalization of patient’s real-time disease trajectory to D50 yields the parameter relative D50 (rD50). The rD50 is an open-ended linear reference scale where 0 signifies disease onset and 0.5 indicates the time-point of halved functionality, providing an individualized time scale of accumulated disease independent of the overall aggressiveness of disease. Patients can be categorized into at least 3 phases: an early semi-stable Phase I (0 ≤ rD50 < 0.25), an early progressive Phase II (0.25 ≤ rD50 < 0.5), and late progressive and late stable Phases III/IV (rD50 ≥ 0.5)

### 2.3. Statistical Analyses

All statistical analyses were performed using the SPSS^®^ software program (v26.0.0.0, IBM^®^, Chicago, IL, USA). Non-normal distribution of all data was established using the Shapiro Wilks test. Therefore, the Mann-Whitney U or the chi-square/Fisher’s exact tests were used to perform between-group comparisons. The longitudinal comparison of patients’ ALSFRS-R scores were conducted with the Wilcoxon signed rank test. *p*-values were adjusted via Bonferroni-correction to account for multiple testing and a statistical significance level of a *p*-value below 0.05 was applied.

The patients were stratified by their individual D50 values, as either having low (D50 < 30 months) or high disease aggressiveness (D50 ≥ 30 months). The cumulative time-dependent probability for reaching a critical event (ventilation or gastrostomy) was estimated using the Kaplan–Meier method and a log-rank test was applied to measure differences between both disease aggressiveness subgroups.

## 3. Results

### 3.1. Triage in the Prospective Cohort

We identified 177 patients who had at least one in person contact in our center since 1^st^ March 2019. Seventy-five patients had highly aggressive ALS (D50 < 30 months) and 20 (26.7%) of them had already died. One hundred and two patients had low disease aggressiveness; within this group, significantly fewer people had died until 1^st^ March 2020 in comparison to the highly aggressive ALS group (*n* = 5, 4.9%; Fischer’s exact test *p* < 0.001). An overview of the demographic and clinical data of alive patients is given in Table 1.

We additionally identified patients who had only one ALSFRS-R assessment because the D50 model carries the highest uncertainty in this group. There were 11 patients with one ALSFRS-R allocated to the low aggressiveness group, seven of whom decided for an active follow-up at our center. Importantly, only one female patient needed a planned intervention with initiation of non-invasive ventilation (NIV) and a gastrostomy in April and at follow-up was allocated to the high aggressiveness group (D50 = 12.5 months).

During the triage of patients for our center we paid specific attention to the high aggressiveness patients. Here, we report all events that occurred between 1^st^ March and 31^st^ May 2020 for this group. Seventeen patients were lost to follow-up (e.g., due to relocation to another city). One patient died 13 months after bulbar onset with highly aggressive ALS disease (D50 = 7.19 months).

**Figure 3:**
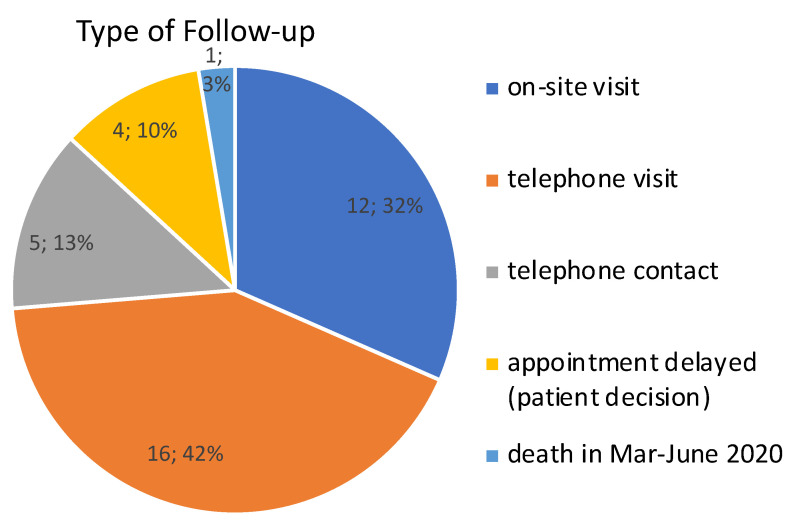
Type of follow-up in the period between 1^st^ March and 31^st^ May 2020 for the 38 patients with highly aggressive ALS.

For the remaining 37 patients we balanced their disease specific needs (e.g., necessity of a sleep study with polygraphy) and their abilities and wishes (e.g., long travel distances, or dependence on relatives/caregivers). The patients were offered pre-chosen options for either on-site or telecommunication-based appointments (Figure 3). The resulting follow-up visits fell into 3 categories and were conducted by a physician with a minimum of two years of experience in ALS care (R.S., A.R., or B.I.). The three categories were: A) on-site visits: including thorough scoring, advanced care planning, clinical examination and a sleep study where necessary; B) telephone-visits: including scoring, counselling, and focused clinical examination (video-assisted where possible); or C) telephone contacts: that were focused on individualized advanced care planning. Only four patients refused the direct offer for any of these opportunities against our specific recommendations, they were encouraged (as before) to call/contact our center whenever necessary. One of these patients actively decided against any further appointment, and the other three patients postponed their appointments until June 2020.

The majority of patients favored telephone contact although we offered real-time video-contact via a telemedicine platform (RED Medical Systems, www.redmedical.de). The latter was successful in only one contact for a high aggressiveness patient. Typical obstacles were disease-related problems (e.g., inability to hold a smart-phone), limited availability of required devices at patients’ home (e.g., smart-phone or computer with video-camera needed), miscellaneous technical constraints (e.g., poor internet bandwidth), and patient refusal.

### 3.2. Outcomes in the Prospective Cohort

**Figure 4:**
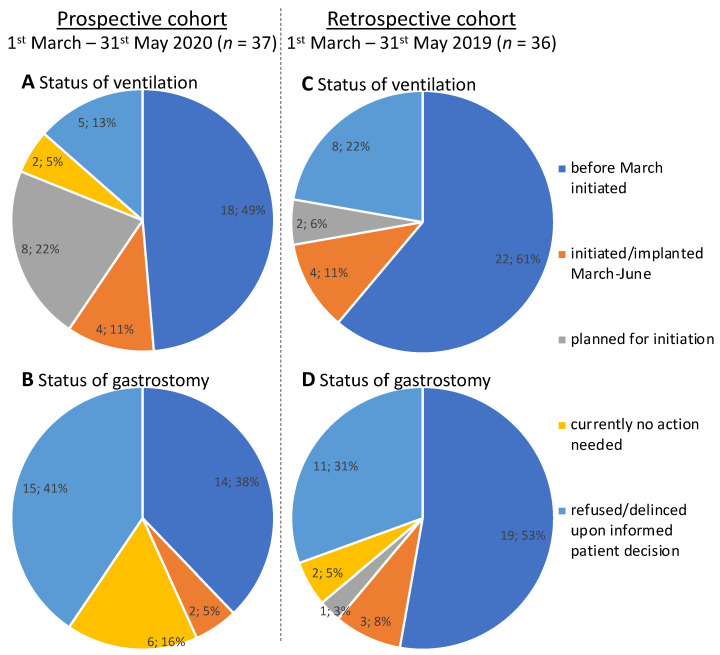
Results of the active follow-up for the prospective cohort from March until June 2020, concerning (**A**) the status of assisted ventilation or (**B**) the status of gastrostomy. For comparison purposes, the same situation is illustrated for the retrospective cohort, regarding the status of (**C**) ventilation and (**D**) gastrostomy.

Within this period, we organized the initiations of NIV for 4 patients with highly aggressive ALS, preparations for another eight patients were undertaken, who were scheduled for June until early July to receive NIV. Precautions for hospitalization were consistently adapted to the latest COVID-19 recommendations given by the German Robert Koch Institute (RKI). All hospitalized patients were negatively tested for an infection with the severe acute respiratory syndrome coronavirus 2 (SARS-CoV-2), before any airway-bound diagnostics or use of a ventilator. Two of the 37 patients in this group received sleep studies shortly before March 2020 which did not indicate a need for assisted ventilation, and they were appropriately planned for follow-ups in the sleep laboratory. Five patients refused a proactive sleep study, all of them were fully informed about the nature and purpose of this therapeutic option as well as possible and negative consequences if rejected or delayed. Three of these patients declared their final decision to reject any kind of assisted ventilation. Neither of these patients had any respiratory symptoms at the time of visit and they were informed to contact our center immediately if such symptoms would occur. The remaining 18 patients were already under treatment with assisted ventilation (three with tracheostomy, 15 NIV, Figure 4A).

For two patients a feeding tube was implanted (one in April and one in May 2020). The proportion of refusal for a gastrostomy was higher for this therapeutic intervention than for assisted ventilation. Fifteen fully informed patients confirmed their will to not have a feeding tube placed at any time during their disease course. For six patients, a feeding tube was considered to be not immediately needed: neither of these patients reported any bulbar symptoms (12 of 12 points in bulbar subscores of the ALSFRS-R) and all had a functional vital capacity above 60% at last measurement. Fourteen patients had already received a gastrostomy prior to March 2020 (Figure 4B).

None of the 37 patients required any emergency medical attendance, unplanned hospitalization or initiation of invasive ventilation (tracheostomy) between 1^st^ March and 31^st^ May. The total ALSFRS-R for the 28 patients who received scoring during their follow-up significantly declined from a median of 34 (interquartile range: 11, 95%-CI: 29–37) to 28 points (interquartile range: 16, 95%-CI: 25–32; *p* < 0.001).

### 3.3. Outcomes in the Retrospective Cohort

We retrospectively identified a cohort of 174 patients who were treated in a comparable time frame before the COVID-19 pandemic (1^st^ March–31^st^ May 2019). Here, 79 of these had highly aggressive ALS, and of them, 54 were still alive on the 1^st^ March 2019. Nine patients were lost to follow up and nine patients died within the regarded time-period.

As in the prospective cohort, NIV therapy was initiated in 4 patients within the specified time period. Two patients were considered as planned for ventilatory support, and had it implemented in June 2019. Eight patients had finally decided against a ventilatory support therapy and a later initiation was not recorded (Figure 4C).

A feeding tube was implanted in three patients and one patient was under preparations, receiving it in July 2019. For two patients, no action was needed at this stage, but both underwent a gastrostomy later in early 2020. The remaining 11 patients had made an informed decision against a gastrostomy and a later initiation of the therapy was not recorded (Figure 4D).

### 3.4. Retrospective Long-Term Analysis Of D50 Subgroups

**Figure 5:**
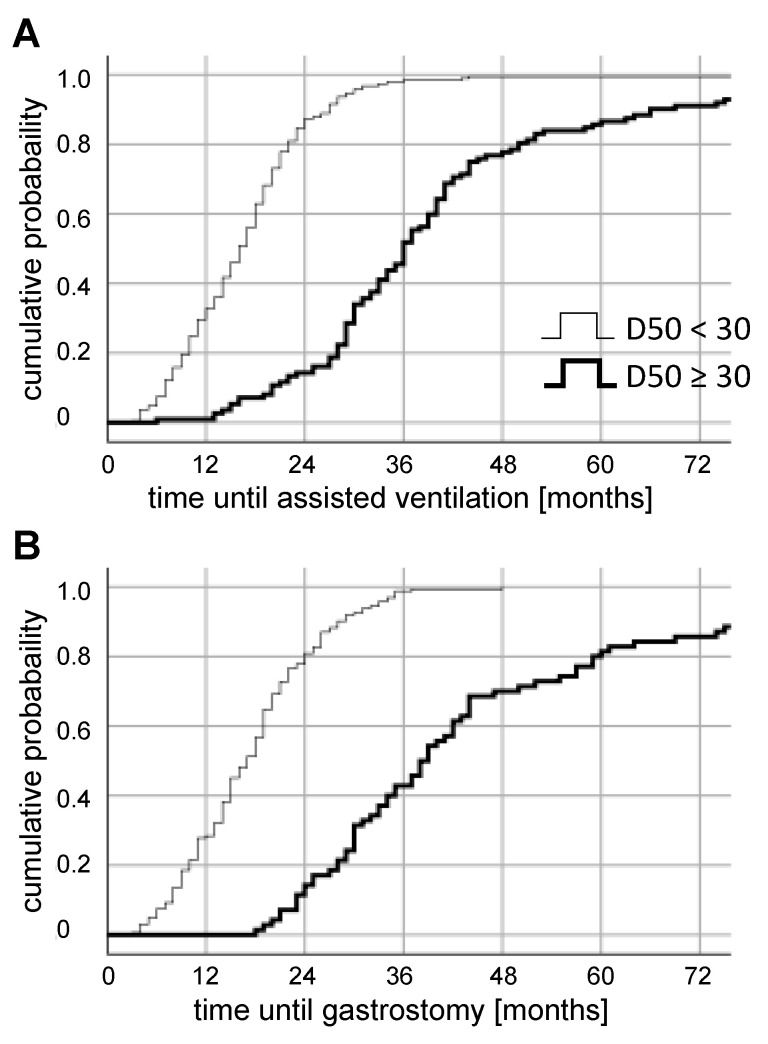
Retrospective stratification of patients via D50 for the cumulative probability of the initiation of therapies after symptom onset. Patients were divided in subgroups of high disease aggressiveness (D50 < 30 months, slim graphs) and low disease aggressiveness (D50 **≥** 30 months, bold graphs). The time is calculated from symptom onset until (**A**) initiation of assisted ventilation (invasive or non-invasive), (**B**) implantation of a feeding-tube. Notably, both events occurred significantly earlier in the group with a lower D50 (Log Rank *p* < 0.001).

Using the entries of 651 ALS cases from our neuromuscular database, we calculated the cumulative probability after symptom onset for a gastrostomy or initiation of ventilation, stratified by D50-subgroups (Figure 5).

Time until first use of assisted ventilation was known for 301 patients. For the subgroup of patients with a D50-value below 30 months, a significantly shorter time until ventilatory support was revealed, with a median of 16 months (95%-CI: 14.5 – 17.5; *p* < 0.001; Figure 5A). For these patients with higher disease aggressiveness, the cumulative probability of assisted ventilation after three months was 0.53%, after six months 7.41%, and 32.8% at 12 months. Lower aggressive patients (D50 **≥** 30 months) had a longer ventilation-free period with a median of 36 months (95%-CI: 33.61–38.39). The cumulative probability for this group was 0% after three months and 0.89% after both six months and 12 months.

Time until gastrostomy was known for 232 patients. The Log Rank test revealed that higher aggressive patients had a significantly shorter time until gastrostomy with a median of 17 months (95%-CI: 15.34–18.66; *p* < 0.001; Figure 5B). Their cumulative probability for the need of a feeding tube was after three months 0.06%, 0.74% after six months, and 2.84% at 12 months. The subgroup of patients with a lower disease aggressiveness had at a median 38 months without gastrostomy (95%-CI: 33.45–42.56), the cumulative risk after 12 months was 0% (the first patient received a feeding tube 18 months after symptom onset).

## 4. Discussion

In this study we report a workflow for triage of ALS patients during the COVID-19 pandemic. For this purpose, we comprehensively evaluated patients’ individual disease aggressiveness via the D50 value. We thus identified a group of patients with higher disease aggressiveness for whom special efforts were undertaken in order to provide them with adequate follow-up. The frequency of care events (NIV and gastrostomies) within the three-month study period were comparable to a retrospective cohort from a comparable time period.

We experienced mostly positive reactions by the patients and/or their relatives and our active follow-up offers were accepted by the majority of patients. We were able to provide and use different mediums to follow up with patients; telephone contact was preferred. This was favored because the transport to our center and related issues could be avoided (our patients travel distances of up to 250 kilometers). Efforts to aid these telephone-visits with a real-time video were only successful on one occasion. Although we are convinced that video-telemedicine is a promising opportunity to provide remote follow-up with patients [21,22], it will not be available for all patients. Obstacles were, e.g., unavailability of technical devices, low internet bandwidth, or patient-specific factors [21,23].

The acute necessity of a triage for the ALS patients due to the COVID-19 pandemic has been reported for other tertiary ALS centers before [24,25,26]. Special attention is required because the infectious disease caused by SARS-CoV-2, is particularly dangerous for ALS patients. It is known that older adults with premorbid chronic disease, especially causing respiratory insufficiency, have a higher mortality/morbidity risk if affected by COVID-19 [27,28]. ALS cohorts typically match these factors (see also Table 1 and Supplementary Table S1), thus an ALS population as a whole can be considered as a typical high-risk group if infected with SARS-CoV-2 [29,30]. Therefore, in-patient and out-patient consultations, patient transportation, or unnecessary contact with other people needed to be reduced to the bare necessity [31,32].

ALS is known to show a high heterogeneity of symptom worsening and patterns of spread throughout the affected regions [33,34]. Thus, nonreflective postponements of consultations, that were impromptu demanded by many patients in March 2020, could cause significant harm or delay supportive therapies. We were convinced, that patients needed differentiated levels of attendance during a special situation like the one caused by the COVID-19 pandemic and defined two principal categories, in our case defined by the D50 model. The first one needed more frequent follow-up, because symptom worsening and/or spread occur remarkably faster than for the patients of the second category. Patients of category 2 were nevertheless not unattended, but postponements of their 3-monthly appointments were more likely accepted if demanded by the patients. The amount of action for high aggressive patients of the retrospective as well as the prospective cohort in terms of the required therapies (NIV or gastrostomy), demonstrated again that the value D50 per se is capable to quantify disease aggressiveness [11,12,13]. The group of patients with higher disease aggressiveness, that includes the category 1 patients in a broad frame, needed regular initiation, preparation, counselling and control of assisted ventilation or gastrostomy in this short reference period of 3 months. The retrospective Kaplan–Meier analyses confirmed that therapeutic interventions in this highly aggressive subgroup occur in significantly shorter time-frames. A nonreflective delay of associated actions (e.g., preparatory measures or counseling) would therefore most likely jeopardize these patients.

Gastrostomy is an established palliative therapy that is known to increase quality of life and to prolong survival for ALS patients [16,17,18,19]. Thereby, consensus guidelines agree that an implantation early in disease increases the benefit for patients [35,36]. A still high-enough forced vital capacity, preferable above 50% of normal range, is a principal requirement intended to determine the right time-point of gastrostomy in the ALS course [17,37]. This was consequently considered during the regular appointments at our center, because it requires early counselling of patients and their relatives, even in stages when no dysphagia is present, in order to foster an informed decision making. In addition, decisions of patients may shift, e.g., as a complex result of their dynamic emotional response to the disease as well as their socio-cultural background [38,39,40]. Thus, a minority of the 15 patients who decided against a gastrostomy, might change their decision. A relevant proportion of ALS patients refusing a gastrostomy was recognized before, in our as well as other ALS centers [17,38]. The group of six patients who were considered not to need a timely initiation of gastrostomy were nevertheless fully informed about this therapy, and a definite decision of these patients is still pending.

Non-invasive ventilation (NIV) is considered a standard palliative therapy that is capable to lower symptom burden and to prolong survival in ALS [14,15]. However, patients usually need to adapt to the NIV, especially when they need to apply it during sleep-times. Therefore, specialized sleep-laboratories should regularly ensure early examinations for the need, the initiation and follow-up controls of effectiveness of a NIV for ALS patients [41,42]. Again, we could provide the patients of our high aggressive prospective group with sufficient information and counselling for this therapeutic option. The proportion of refusal for a NIV (five of 37 patients) was noticeably smaller than compared to decisions against a gastrostomy. Hence, half of them were already initiated for this therapy and more than 75% will be under therapy and thus probably benefit. Altogether, we are convinced that we were able to offer the necessary patient-centered advanced care planning in terms of gastrostomy as well as NIV during the initial phase of the pandemic.

The consideration of high-risk patients under active follow-up at our center was promptly possible due to the availability of an established and specialized database. Regular entries of patient-specific data were long before integrated in daily clinical routines at our center and are based on clinical milestones in the framework of the D50 model in order to enable the management of multidisciplinary therapies. The advantages of organizing patient’s disease-specific information in a database were postulated before, especially in the context of (future) ALS research [43,44]. Here, we show additional utility of the D50 model to foster and maintain quality of advanced care planning for ALS patients. The model based categorization will be utilized in all motoneuron and neuromuscular disease patients as the ongoing pandemic challenges all comprehensive care in patients with high, intermediate, and low aggressive neuromuscular disease.

### Limitations

The present study is not without its limitations. To begin with, we cannot provide direct evidence, that our approach directly prevented poorer outcomes for our patients. This assessment will become available with long-term clinical milestone data (e.g., survival). Naturally, telephone contacts with patients didn’t allow for detailed neurological examinations or diagnostic workups; spirometry and cough flow assessments were not possible, thus important clinical information may have been missed. However, COVID-19-related restrictions began to be lifted from June 2020 onwards, which allowed us to see more patients on-site within the framework of our three-monthly comprehensive follow-ups. Thus far, our clinical data strongly suggest that our approach successfully helped to mitigate the healthcare disruptions caused by the three month “lockdown” period and met the needs of patients with highly aggressive forms of ALS.

The data presented here are mono-centric and therefore cannot be generalized for ALS patients, e.g., due to pre-existing referral bias that is typically observed in tertiary centers [45]. Also, equipment and abilities, location factors (e.g., referral area), and patients characteristics can relevantly differ in other ALS centers.

Finally, possibly contributing patient-specific co-factors were incompletely known, e.g., the access to allied health care services, comorbidities or cognitive impairment. The patients of this study were in different stages of the diagnostic workup in the beginning of the pandemic, thus, e.g., standardized neuropsychological testing was not available for all of them. Further multi-center studies incorporating information about patients’ access to allied health care are needed in order to provide implications for possible future lockdown situations.

## 5. Conclusions

We were able to highlight the ability of the D50 model to identify ALS patients with higher disease aggressiveness. These patients need particular attention and frequent follow-ups, especially during periods of restricted access to health resources like the COVID-19 pandemic. Based on this, we demonstrate how patient triage in combination with anticipatory counselling help to balance their high dependence on the timely initiation of therapies whilst minimizing their risk of infections with the novel coronavirus SARS-CoV-2 (e.g., due to unnecessary hospitalizations).

The implementation of the workflow presented in this study and the D50 model itself is feasible and can be recommended for other (tertiary) ALS centers in order to maintain quality standards for advanced care planning.

## Figures and Tables

**Table 1:** Demographic and Clinical Data for alive patients of the prospective cohort, stratified by D50.

	High Aggressiveness (D50 < 30)	Low Aggressiveness (D50 ≥ 30)	*p*
*n*	55	97	
**Age** At Symptom Onset [In Years]	65.3 ± 13.6 (62.3 – 67.6)	59.6 ± 15.0 (57.8 – 62.3)	0.015*
**Symptom Duration** In Mar-2020 [In Months]	20.0 ± 18.0 (17 – 27)	46.0 ± 39.0 (40 – 54)	<0.001*
**rD50** In Mar-2020	0.55 ± 0.52 (0.46 – 0.69)	0.34 ± 0.36 (0.28 – 0.39)	<0.001*
**Gender** [Male/Female]	26/29; 47.3%/52.7%	56/41; 57.7%/42.3%	1
**Onset-Type** [Bulbar/Limb/Generalized/ Respiratory]	22/29/1/3; 40.0%/52.7%/1.8%/5.5%	16/79/1/1; 16.5%/81.4%/1%/1%	0.005*
**Phase** In Mar-2020 [I/II/III&IV]	1/24/30 1.8%/43.6%/54.5%	32/36/59 33%/37.1%/29.9%	<0.001*
Prior **Assisted Ventilation** In Mar-2020 [No/Yes]	32/23; 58.2%/41.8%	59/38; 60.8%/39.2%	0.191
Prior **Gastrostomy** In Mar-2020 [No/Yes]	31/24; 56.4%/43.6%	81/16; 83.5%/16.5%	0.002*

Metric variables are presented as median ± interquartile-range and 95%-Confidence-Intervals of the median; categorial variables are presented as number and percentage (see also Supplementary Table S1). * Asterisks mark significant differences in-between groups at *p* < 0.05 (after Bonferroni-correction).

## Supplementary Materials

The following are available online at https://www.mdpi.com/2077-0383/9/9/2873/s1, Table S1: Demographic and Clinical Data for the entire prospective cohort, stratified by D50.
