# Supplementary material for: Triage of Amyotrophic Lateral Sclerosis Patients during the COVID-19 Pandemic: An Application of the D50 Model
Source: J Clin Med. 2020 Sep 5;9(9):2873. doi: 10.3390/jcm9092873 (PMC7565659; doi:10.3390/jcm9092873)
Supplement: Supplementary file 1 [file jcm-09-02873-s001.pdf]

Supplementary material

Table S1. Demographic and Clinical Data for the entire prospective cohort, stratified by D50.

	**high aggressiveness (D50 < 30)**	**low aggressiveness (D50 ≥ 30)**	***P***
n	75	102	
**Age** at symptom onset [in years]	65.8 ± 15.3 (63.3 - 69.8)	60.1 ± 14.6 (58.1 - 62.8)	<.001*
**Gender** [male/ female]	37/ 38; 49.3%/ 50.7%	59/ 43; 57.8%/ 42.2%	.287
prior **Assisted ventilation** in Mar-2020 [no/ yes]	33/ 42; 44.0%/ 56.0%	60/ 42; 58.8%/ 41.2%	.067
symptom **duration until ventilation** [months]	16 ± 10 (14 - 19)	39 ± 23 (36 - 49)	<.001*
prior **Gastrostomy** in Mar-2020 [no/ yes]	40/ 35; 53.3%/ 46.7%	83/ 19; 81.4%/ 18.6%	<.001*
symptom **duration until Gastrostomy** [months]	16 ± 15 (11 - 23)	42 ± 43 (34 - 75)	<.001*
prior **Death** in Mar-2020 [no/ yes]	55/ 20; 73.3%/ 26.7%	97/ 5; 95.1%/ 4.9%	<.001*
symptom **duration until Death** [months]	22 ± 19 (15 - 29)	44 ± 50 (42 - 93)	<.001*
**time since last ALSFRS-R** [months]	4 ± 7 (3 - 6)	3 ± 5 (3 - 4)	.011*
**ALSFRS-R** [points]	26 ± 20 (21 - 31)	37 ± 19 (33 - 40)	<.001*
**ALSFRS-R bulbar subsc.**	7 ± 8 (5 - 10)	11 ± 3 (11 - 12)	<.001*
**ALSFRS-R fine-motor subsc.**	5 ± 7 (4 - 7)	8 ± 7 (8 - 9)	<.001*
**ALSFRS-R gross-motor subsc.**	5 ± 7 (4 - 7)	7 ± 7 (6 - 9)	.005*
**ALSFRS-R ventilatory subsc.**	9 ± 6 (8 - 11)	11 ± 5 (11 - 12)	.003*
**King's stage** [I/ II/ III/ IVa / IVb/ V]	5/ 10/ 12/ 14/ 14/ 20; 6.7%/ 13.3%/ 16.0%/ 18.7 %/ 18.7%/ 26.7%	17/ 31/ 16/ 7/ 26/ 5; 16.7%/ 30.4%/ 15.7%/ 6.9 %/ 25.5%/ 4.9%	<.001*
**MiToS stage** [0/ I/ II/ III/ IV/ V]	19/ 14/ 8/ 8/ 6/ 20; 25.3%/ 18.7%/ 10.7%/ 10.7%/ 8.0 %/ 26.7%	56/ 16/ 19/ 5/ 1/ 5; 54.9%/ 15.7%/ 18.6%/ 4.9%/ 1.0 %/ 4.9%	<.001*

Metric variables are presented as median ± interquartile-range and 95%-Confidence-Intervals of the median, the *P*-values (uncorrected) refer to two-sample Mann-Whitney-U-tests. Categorial variables are presented as number and percentage, the *P*-Values refer to chi-square or Fischer-exact-tests were appropriate. For comparison purpose, traditionally used staging systems are given, as calculated from the latest available ALSFRS-R score, the time until last ALSFRS-R is calculated until the 1st March 2020. The King's Staging system allocates patients to Stages I (involvement of one clinical region) until IV (respiratory or nutritional failure), whilst the MiToS System describes Stages 0 (functional involvement) until IV (loss of independence in four domains), the Stages V depict patients' death respectively (Balendra et al., 2014; Chio, Hammond, Mora, Bonito, & Filippini, 2015; Roche et al., 2012).

Abbreviations: ALS = Amyotrophic Lateral Sclerosis; ALSFRS-R = ALS Functional Rating Scale (revised), with bulbar subscore-sums of items 1-3, with fine-motor subscore-sums of items 4-6, with gross-motor

subscore-sums of items 7-9 and with ventilatory subscore-sums of items 10-12; MiToS = Milano-Torino Staging.

References:

Balendra, R., Jones, A., Jivraj, N., Knights, C., Ellis, C. M., Burman, R., . . . Al-Chalabi, A. (2014). Estimating clinical stage of amyotrophic lateral sclerosis from the ALS Functional Rating Scale. *Amyotroph Lateral Scler Frontotemporal Degener, 15*(3-4), 279-284. doi:10.3109/21678421.2014.897357

Chio, A., Hammond, E. R., Mora, G., Bonito, V., & Filippini, G. (2015). Development and evaluation of a clinical staging system for amyotrophic lateral sclerosis. *J Neurol Neurosurg Psychiatry, 86*(1), 38-44. doi:10.1136/jnnp-2013-306589

Roche, J. C., Rojas-Garcia, R., Scott, K. M., Scotton, W., Ellis, C. E., Burman, R., . . . Al-Chalabi, A. (2012). A proposed staging system for amyotrophic lateral sclerosis. *Brain, 135*(Pt 3), 847-852. doi:10.1093/brain/awr351
